# Prevalence of polypharmacy use and association with mortality: a cohort study of elderly people in Southern Brazil, 2014-2017

**DOI:** 10.1590/S2237-96222025v33e20240081.en

**Published:** 2025-04-07

**Authors:** Cristina Heloisa Müller, Andréa Dâmaso Bertoldi, Renata Moraes Bielemann, Karla Pereira Machado, Elaine Tomasi, Maria Cristina Gonzalez, Marysabel Pinto Telis Silveira

**Affiliations:** 1 Universidade Federal de Pelotas, Instituto de Fisiologia, Programa de Pós-Graduação Multicêntrico em Ciências Fisiológicas, Pelotas, RS, Brazil; 2 Universidade Federal de Pelotas, Faculdade de Medicina, Programa de Pós-Graduação em Epidemiologia, Pelotas, RS, Brasil; 3 Universidade Federal de Pelotas, Faculdade de Nutrição, Programa de Pós-Graduação em Nutrição e Alimentos, Pelotas, RS, Brasil

**Keywords:** Polypharmacy, Elderly, Aging, Mortality, Cohort studies, Polifarmacia, Anciano, Envejecimiento, Mortalidad, Estudios de Cohortes

## Abstract

**Objective:**

To characterize prevalence of polypharmacy use and its association with mortality in elderly people.

**Methods:**

Prospective cohort (2014 to 2017), with non-institutionalized individuals aged 60 years or over, living in Pelotas, Rio Grande do Sul, Brazil. Association between polypharmacy and mortality was analyzed using Cox regression. Hazard ratios (HR) and their respective 95% confidence intervals (95%CI) were calculated, following the Cox proportional hazards model. Interaction between age group

and multimorbidity was considered to be statistically significant when p-value&lt;0.100.

**Results:**

Results: Polypharmacy prevalence was 36.1% (95%CI 33.7; 38.6), being higher as age group increased (29.8% in those aged 60-69; 41.3% in those aged 70-79). and 47.8% in

those aged 80 years and over). In the adjusted analysis, risk of mortality was 62% higher among elderly people using polypharmacy (HR 1.62; 95%CI 1.10; 2.39), without interaction between age group (p-value 0.750) and multimorbidity (p-value 0.312). The

survival analysis demonstrated that the probability of survival was lower in elderly people using polypharmacy (85.6%).

**Conclusion:**

Polypharmacy prevalence was found to be higher as age group increased and elderly people using polypharmacy had higher risk of mortality, regardless of age group and presence of multimorbidity.

## Resumen

**Objetivo:**

Caracterizar la prevalencia del uso de polifarmacia y su asociación con la mortalidad en ancianos.

**Métodos:**

Cohorte prospectiva (2014 a 2017), con personas no institucionalizadas, de 60 años o más, residentes en Pelotas, Rio Grande do Sul, Brasil. La asociación entre polifarmacia y mortalidad se analizó mediante regresión de Cox. Se calcularon los índices de riesgo (HR) y sus respectivos intervalos de confianza del 95% (IC95%), siguiendo el modelo de riesgos proporcionales de Cox. La interacción entre grupo de edad y multimorbilidad se consideró estadísticamente significativa con valor de p-valor &lt;0,100.

**Resultados:**

La prevalencia de polifarmacia fue del 36,1% (IC95% 33,7; 38,6), siendo mayor a medida que aumentó el grupo de edad (29,8% en los de 60 a 69 años; 41,3% en los de 70 a 79 años y 47,8% en el grupo de edad de 80 años y más). En el análisis ajustado, el riesgo de mortalidad fue 62% mayor entre los ancianos que usaban polifarmacia (HR 1,62; IC95% 1,10; 2,39), sin interacción entre grupo de edad (p-valor 0,750) y multimorbilidad (p-valor 0,312). En el análisis de supervivencia se demostró que la probabilidad de supervivencia era menor en aquellos ancianos con polifarmacia (85,6%).

**Conclusión:**

Se identificó que la prevalencia de la polifarmacia era mayor a medida

que aumentaba el grupo de edad y que los ancianos con polifarmacia tuvieron mayor riesgo de mortalidad, independientemente del grupo etario y de la presencia de multimorbilidad.

**Ethical aspects**

This research respected ethical principles, having obtained the following approval data:

Research ethics committee: Universidade Federal de Pelotas

Opinion number: 472.357

Approval date: 28/11/2013

Certificate of submission for ethical appraisal: 54141716,0.0000,5317

Consent form: Obtained from all participants before telemedicine consultation.

## Introduction

Use of medications by elderly people, despite the risks, is of paramount importance not only as a pharmacological strategy to reduce the process of degradation involved in aging, but also for the control of chronic diseases ^1^. If, on the one hand, individuals are living longer due to discoveries of new medications, on the other hand, there is the potential risk of drug interactions and adverse effects due to the use of multiple medications, which is becoming one of the main causes of admission to health services and a major public health problem ^2^.

New studies to ensure the safety of pharmacotherapy are essential, especially due to the complexity of aging and use of multiple medications, as well as the indisputable correlation with survival time as age increases. Considering that elderly people, according to the 2022 Demographic Census, represent an important portion of the Brazilian population (15.8%) ^3^, and that use of medication is more frequent in this age group, this issue becomes extremely relevant.

A systematic review, covering the period from 2000 to 2016, studied the term polypharmacy in order to identify and summarize its definitions in the existing literature ^4^. That review highlights the challenges for definition given the variability in the numerical limit, as well as inconsistencies regarding duration of therapy and use of over-the-counter medications ^4^. Most studies define polypharmacy as the concomitant use of five or more medications ^5^ ^,^ ^6^. The limitation of the literature, as well as comparison between studies, becomes difficult due to the variation in methodological aspects, including the definition of polypharmacy, age groups and follow-up time.

An 18-year follow-up study carried out in England and Wales reports that polypharmacy is associated with increased short-term mortality in elderly people, regardless of sex, age, smoking status, institutionalization, disability as to daily living activities and health conditions. That study noted that it remains nuclear whether polypharmacy is a marker for poor health or is an independent risk factor for mortality, having been described as a predictor of adverse drug outcomes ^7^. The objective of this study was to characterize prevalence of polypharmacy use and its association with mortality in elderly people.

## Methods

### Study design

Prospective cohort study (2014 to 2016 and 2017). 

### Background

**Figure 1:**
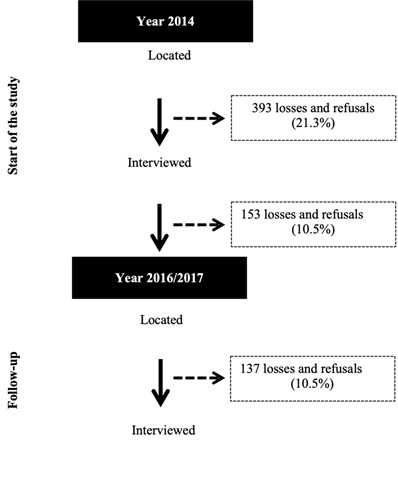
Number of participants in each stage of the study.Pelotas, Brazil, 2014-2017

The study began in 2014, with 1,451 elderly people who after being located were interviewed at home. It began as a cross-sectional study, but in 2016 the decision was taken to continue it, and it gained the name: “Longitudinal Study of Elderly People’s Health” - “COMO VAI?” (HOW ARE YOU?), starting the second stage of follow-up with telephone and home interviews, whereby 1,161 elderly people were interviewed (Figure 1). The phone calls were made on different days and times. When it was not possible to make telefone contact, at least four attempts were made to find the elderly people at their addresses. This planning was important for confirming basic information, enabling deaths to be identified on the Mortality Information System (Sistema de Informação sobre Mortalidade).

### Participants

Participants were individuals aged 60 years or over, non-institutionalized, living in the urban area of Pelotas, Rio Grande do Sul, Brazil.

### Variables

The main exposure variable was polypharmacy,characterized as the use of five or more medications ^5^,which had been measured in the initial assessment (2014).

The outcome was mortality, the observation time used to estimate it was from the date of the interview in 2014 until death or the end of the follow-up period (2016/2017).

### Data source and measurement

In order to check for use of medications, the following questions were asked: Do you need to take any long-term medication? and: Have you taken any medication in the last 15 days?.

Death due to all causes was taken into consideration,whereby this information was provided in telefone or home interviews, by a relative or neighbor, and was later checked with the Epidemiological Surveillance Sector of the Pelotas Municipal Health Department, by means of the Mortality Information System (Sistema de Informação sobre Mortalidade) and by family members showing death certification documents. In the analysis, people who refused to participate at this stage were considered to be alive, in the same way as individuals who were classified as losses to follow-up due to their not being able to be contacted, but for whom there were no death records, were also considered to be alive.

### Bias control

In order to minimize memory bias, prescriptions and medication packaging were requested and questions were asked about long-term medications, in addition to those used in the last 15 days.The elderly people who were lost to follow-up in the study were taken proportionally over time, thus being considered a more conservative position for calculating hazard ratio (HR) estimates. In other words, when measuring a person’s risk over time, we considered up to half of their total follow-up time, as there was no way of knowing the exact moment between one visit and another when the elderly person became lost to follow-up. Strategies were adopted to duly identify deaths that occurred among participants.

### Study size

The sample size was estimated for the “Research Consortium” carried out by the Postgraduate Program in Epidemiology at the Universidade Federal de Pelotas ^8^. In 2014, a larger sample was estimated necessary to achieve the study objectives of each master’s degree student, considering an additional 15% for confounding factors and 10% for losses and refusals, in addition to the sample design effect (1.5%), totaling 1,649 ^9^. For the master’s research aiming to identify the use of inappropriate medications for elderly people, the total sample size was 1,646 subjects ^9^ ^,^ ^10^. The sampling process was carried out in two stages. Firstly, the clusters identified in the 2010 Census were chosen ^11^. Of the 488 census tracts, 469 were eligible, and tracts with a small number of elderly people compared to the others were grouped together. In the selection process, the tracts were ordered according to average income, ensuring the inclusion of all neighborhoods and different economic status. Each tract contained information on the number of households, by initial number and final number, totaling 107,152 households. Based on the 2010 Census, in order to achieve the sample size of 1,649 individuals, 3,745 households needed to be included. In the second stage, census tracts were drawn in order to select households. Thirty-one households were selected per tract, enabling the identification of at least 12 elderly people in each one, so that 133 census tracts were included ^9^.

### Quantitative variables

The independent variables included demographic and socioeconomic data, lifestyle data and data related to health conditions: age group (in years: 60-69; 70- 79; 80 or over); race/skin color observed (White; non-White); sex (male; female); schooling (in years of study: none; less than 4; 4-10; 11 or more); economic classification according to the Brazilian Association of Research Companies ^12^ grouped into (A/B; C; D/E); marital status (married/has partner; single/ separated, widowed/no partner); and, current work status (working; not working). Lifestyle variables included tobacco smoking (no, never smoked; yes, has smoked 1 or more cigarette(s) per day for more than 1 month; used to smoke, but has stopped smoking) and alcoholic beverage consumption in the last 30 days (no; yes). The variables related to health conditions were: a) self-perceived health, by asking the question: How do you rate your health? (good, very good; regular; poor, very poor); b) multimorbidity (no; yes), defined as the presence of two or more chronic conditions ^13^ by means of asking the question Has any doctor or health professional ever said that you have (...) ? whereby 28 diseases or symptoms are listed; c) type of health service (private or insurance/Brazilian National Health System; Sistema Único de Saúde - SUS) by asking: where did you last consult in the last year? and was the care in the health service where you last consulted provided through health insurance, private services or through the SUS?.Statistical methods The statistical analyses were performed using Stata software version 17 (StataCorp, College Station). Initially, a descriptive analysis of the total sample and polypharmacy prevalence was performed according to sociodemographic, lifestyle and health conditions variables, using chi-square or linear trend tests. Subsequently, polypharmacy prevalence was analyzed according to the variables studied, stratified by age group, presenting the proportions and respective 95% confidence intervals (95%CI), using the chi-square test. Hazard ratios (HR) and their respective 95%CI were calculated in order to evaluate the effect of polypharmacy on mortality, following the Cox proportional hazards model, using Cox regression. Statistical adjustment was performed by including the main exposure variable (polypharmacy) and the outcome (mortality) based on a hierarchical model with four levels, where level 1 comprised sociodemographic and economic variables, level 2 comprised lifestyle variables and level 3 comprised health conditions. Only variables with p-value <0.200 were kept in the final model. Following this, Kaplan-Meier survival analysis was performed and the cumulative risk function was plotted according to time to assess whether the results could be influenced by the time between events. The Log-Rank test was used to compare survival functions between individuals with and without polypharmacy. The age group and multimorbidity variables were assessed as effect modifiers of association between polypharmacy and mortality, taking a p-value <0.100 as statistically significant. After adjustment, interaction was tested by removing the “age group” variable from the model in order to assess the possible influence of the interaction. Following this, the variable was reintegrated into the adjusted model.The individuals who participated in the research or their guardians were informed about the study and signed the informed consent form, and data confidentiality was guaranteed. In cases of death in 2016/2017, the consente form was signed by family members or informants. In the case of telephone interviews, consent to answering the questionnaire was verbal.

## Results

In 2014, 1,844 elderly people were located. After 393 losses and refusals (21.3%), 1,451 were included. Among these 1,451, there were 153 (10.5%) losses and refusals, so that 1,298 elderly people were located in the second follow-up (2016/2017). Of these, 1,161 were interviewed, due to a further 137 (10.5%) losses and refusals (Figure 1). Table 1 describes the analysis of the total sample according to sociodemographic, lifestyle and health condition variables. Polypharmacy prevalence for the overall sample was 36.1% (95%CI 33.7; 38.6), being higher as age increased (29.8% in those aged 60-69; 41.3% in those aged 70-79 and 47.8% in those aged 80 years or over), and higher in those of White race/skin color (37.7%), females (39.3%), those who were not working (38.9%), former smokers (39.5%), those who had not consumed alcohol in the last 30 days (38.4%), those with poorer self- perceived health (56.1%), those who had multimorbidity (37.4%) and those who used private health services or health insurance (42.1%) (Table 1).

**Table 1:** Description of the total sample and polypharmacy prevalence according to sociodemographic, lifestyle and health condition variables. Pelotas, 2014-2017 (n=1.451)

Variable	Sample	Polypharmacy	Polypharmacy
	n (%)	% (95%CI)	p-value
**Age group (years)**_a_			<0.001
60-69	756 (52.3)	29.8 (26.6; 33.1)	
70-79	460 (31.8)	41.3 (36.9; 45.9)	
≥80 230	(15.9) 47.8	(41.4; 54.3)	
**Race/skin color**_a_			0.014
White	1,211 (83.7)	37.7 (35.0; 40.4)	
Non-white	236 (16.3)	29.2 (23.8; 35.4)	
**Sex**			0.001
Male	537 (37.0)	30.9 (27.1; 35.0)	
Female	914 (63.0)	39.3 (36.2; 42.5)	
**Schooling (years)**_a_			0.856
None	196 (13.6)	35.7 (29.3; 42.7)	
< 4	337 (23.5)	37.7 (32.7; 43.0)	
4-10	588 (40.9)	36.6 (32.8; 40.5)	
≥11	316 (22.0)	34.5 (29.4; 39.9)	
**Economic class**_a_			0.065
A/B	483 (35.2)	36.0 (31.9; 40.4)	
C	720 (52.5)	37.4 (33.9; 41.0)	
D/E	169 (12.3)	27.8 (21.5; 35.1)	
**Marital status**_a_			0.130
Married/has partner	763 (52.7)	34.5 (31.2; 37.9)	
Single/separated/widowed/no partner	684 (47.3)	38.3 (34.7; 42.0)	
**Work status**_a_			<0.001
Working	264 (19.6)	24.2 (19.4; 29.8)	
Not working	1,084 (80.4)	38.9 (36.1; 41.9)	
**Tobacco smoking**_a_			<0.001
No, never smoked	781 (54.0)	37.5 (34.2; 41.0)	
Yes, has smoked 1 or more cigarette(s) per day for more than 1 month	182 (12.6)	22.5 (17.0; 29.2)	
Used to smoke, but has stopped smoking	483 (33.4)	39.5 (35.3; 44.0)	
**Alcoholic beverage consumption**_a_			0.002
No	1,138 (78.8)	38.4 (35.6; 41.3)	
Yes	307 (21.3)	28.7 (23.9; 34.0)	
**Self-perceived health**_a_			<0.001
Good/very good	765 (53.1)	26.1 (23.1; 29.4)	
Regular	545 (37.8)	45.5 (41.4; 49.7)	
Poor/very poor	132 (9.2)	56.1 (47.4; 64.3)	
**Multimorbidity**_a_			<0.001
No	89 (6.7)	6.7 (3.0; 14.3)	
Yes	1,250 (93.4)	37.4 (34.8; 40.2)	
**Type of health service**_a_			0.029
Private or insurance	812 (63.4)	42.1 (38.8; 45.6)	
Brazilian National Health System	468 (36.6)	35.9 (31.7; 40.4)	

Note: aVariables with missing data: age group (n=5), race/skin color (n=4), schooling (n=14), economic class (n=79), marital status (n=4), work status (n=103), tobacco smoking (n=5), alcoholic beverage consumption (n=6), self-perceived health (n=9), multimorbidity (n=112), type of health service (n=171).

Table 2 describes the prevalence rates of polypharmacy with their respective confidence intervals, stratified by age group, for each of the variables studied. In the 60-69 age group, polypharmacy prevalence was higher in females (33.4%), those in socioeconomic class C (33.2%), those who were not working (32.9%), those who had not consumed alcoholic beverages in the last 30 days (31.7%), those who had poor or very poor self-perceived health (56.5%) and those who had multimorbidity (31.8%). In those aged 70-79, polypharmacy prevalence was higher in females (45.8%), those with self-perceived health reported as poor or very poor (61.4%) and those who had multimorbidity (42.2%). Prevalence was also higher among those who did not have a partner (46.4%), who never smoked (44.8%) and those who used private health services or health insurance (49.6%) (Table 2). Among the elderly aged 80 or over, polypharmacy prevalence was higher in those of White race/skin color (51.1%), with 11 or more years of schooling (56.8%), those with multimorbidity (47.5%) and those who used private health services or health insurance (55.2%) (Table 2).

**Table 2:** Polypharmacy prevalence according to sociodemographic, lifestyle and health condition variables, stratified by age group. Pelotas, 2014-2017 (n=1,451)

Variables	60-69 years		70-79 years		≥80 years	
	% (95%CI)	p-value		p-value		p-value
**Race/skin color**		0.056		0.578		0.043
White						
Non-white						
**Sex**		0.005		0.011		0.378
Male	23.9 (19.3; 29.2)		33.7 (27.0; 41.2)		52.0 (40.6; 63.2)	
Female	33.4 (29.3; 37.8)		45.8 (40.1; 51.6)		45.8 (38.1; 53.8)	
**Schooling (years)**		0.482		0.648		0.043
None	21.7 (12.9; 34.1)		46.1 (35.1; 57.4)		36.7 (25.3; 49.7)	
<4	32.5 (25.6; 40.3)		42.9 (34.2; 52.0)		41.0 (29.2; 53.9)	
4-10	30.2 (25.5; 35.3)		40.3 (33.4; 47.7)		58.0 (45.9; 69.2)	
≥11	29.6 (23.6; 36.4)		36.6 (26.8; 47.7)		56.8 (40.1; 72.0)	
**Economic class**_a_		0.007		0.579		0.166
A/B	27.4 (22.4; 33.0)		43.0 (34.6; 51.8)		55.7 (44.5; 66.4)	
C	33.2 (28.6; 38.2)		41.1 (35.0; 47.3)		43.3 (34.0; 53.0)	
D/E	15.5 (8.7; 26.1)		35.0 (23.9; 48.1)		40.5 (25.7; 57.3)	
**Marital status**		0.509		0.042		0.398
Married/has partner	30.7 (26.6; 35.1)		37.1 (31.3; 43.2)		52.5 (39.8; 64.8)	
Single/separated/widowed/no partner	28.4 (23.6; 33.8)		46.4 (39.7; 53.2)		46.2 (38.7; 53.8)	
**Work status**		0.003		0.081		0.453
Working	21.6 (16.6; 27.7)		28.2 (16.0; 44.7)		58.3 (27.8; 83.6)	
Not working	32.9 (28.9; 37.1)		42.6 (37.8; 47.6)		47.2 (40.2; 54.2)	
**Tobacco smoking**		0.060		0.007		0.289
No, never smoked	29.4 (25.0; 34.3)		44.8 (38.8; 50.9)		45.8 (37.8; 54.1)	
Yes, has smoked 1 or more cigarette(s) per day for more than 1 month	22.2 (15.8; 30.4)		19.0 (9.6; 34.2)		35.7 (14.3; 65.0)	
Used to smoke, but has stopped smoking	34.0 (28.4; 40.1)		41.5 (34.1; 49.4)		54.9 (43.1; 66.2)	
**Alcoholic beverage consumption**		0.046		0.272		0.181
No	31.7 (28.0; 35.7)		42.5 (37.6; 47.6)		49.8 (42.8; 56.7)	
Yes	24.1 (18.5; 30.7)		36.0 (26.5; 46.8)		36.7 (21.1; 55.7)	
**Self-perceived health**		<0.001		<0.001		<0.001
Good/very good	22.0 (18.4; 26.2)		28.2 (22.7; 34.4)		40.0 (30.8; 50.0)	
Regular	36.9 (31.1; 43.0)		51.9 (44.7; 59.0)		55.9 (46.0; 65.3)	
Poor/very poor	56.5 (43.7; 68.4)		61.4 (46.0; 74.8)		48.0 (28.8; 67.8)	
**Multimorbidity**_b_		<0.001		0.001		0.021
No	6.9 (2.6; 17.3)		4.8 (0.6; 29.8)		10.0 (1.0; 54.7)	
Yes	31.8 (28.3; 35.4)		42.2 (37.5; 47.0)		47.5 (40.2; 54.9)	
**Type of health service**		0.251		0.006		0.013
Private or insurance	31.9 (27.4; 36.7)		49.6 (43.7; 55.6)		55.2 (47.2; 62.9)	
Brazilian National Health System	36.2 (30.6; 42.1)		35.5 (28.0; 43.8)		35.7 (24.1; 49.3)	

We identified 145 deaths (10%). Follow-up time was 2.6 (+/-0.5) years. The results of the crude and adjusted Cox proportional hazard regression models are shown in Table 3. Polypharmacy remained a risk factor for death after adjusting for sex, age group, schooling, marital status, work status, tobacco smoking,alcoholic beverage consumption and self-perceived health, increasing the risk of mortality by 62% (HR 1.62; 95%CI 1.10; 2.39). No evidence of effect modification was observed for age group (p-value 0.750) or for multimorbidity (p-value 0.312).

**Table 3:** Crude and adjusted hazard ratio (HR) and confidence interval (95%CI) of polypharmacy by study variables. Pelotas, 2014-2017 (n=1,451)

Variable	Crude analysis		Adjusted analysis	
	HR (IC95%)	p-value	HR (IC95%)	p-value
Polypharmacy				
Entire sample		<0,001		0,014
No	1,00		1,00	
Yes	2,07 (1,48; 2,89)		1,62 (1,10; 2,39)	

**Figure 2.**
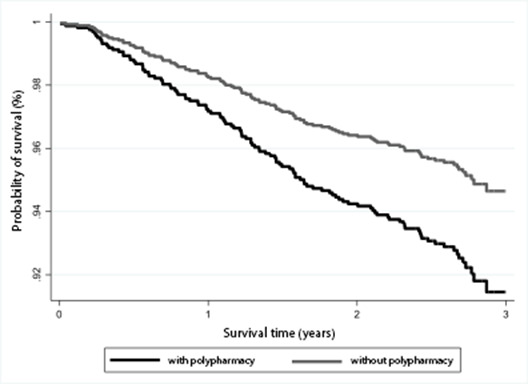
Cumulative survival probability according to polypharmacy in elderly people. Pelotas, 2014-2017 (n=1,451)

Figure 2 shows that both survival curves decrease over time, that is, the probability of survival decreased as age increased, both for elderly people with polypharmacy and for those without polypharmacy. However, the survival probability, after three years, of individuals with polypharmacy at initial assessment was lower (85.6%) than that of individuals without polypharmacy (92.6%). There was no significant difference in the crude analysis (p-value 0.055, log-rank test) but there was a difference in the adjusted analysis (p-value 0.014).

## Discussion

The results demonstrate that polypharmacy is independently associated with mortality in elderly people. Hypothetically, we believed that the risk of mortality with polypharmacy would be greater in the elderly in the oldest age groups, as they would be more likely to have more diseases ^14^ and, consequently, use more medications. However, polypharmacy decreased the probability of survival regardless of age group. This result is similar to that of a previous study that analyzed mortality associated with polypharmacy. Survival of elderly people exposed to polypharmacy was assessed in Brazil for the period 2006-2010 ^15^, demonstrating that probability of survival after five years for individuals with polypharmacy was 77.2%, while for individuals without polypharmacy it was 85.5% (p-value <0.001). Also in that study, the survival curve shows that the probability of death was higher in elderly people with polypharmacy throughout follow-up ^15^.

One of the possible limitations of our study is the relatively short follow-up time. However, the cohort study continues, which will allow mortality to be assessed over a longer period of time in the future. Another limitation is the possible occurrence of memory bias, as it was necessary for participants to remember the medications used in the last 15 days. This bias was minimized by requesting prescriptions and medication packaging and also asking about long-term medication. In our study, multimorbidity did not modify the effect of association between polypharmacy and mortality. Polypharmacy prevalence was significantly higher when multimorbidity was present, in all age groups, increasing with advancing age. A cohort study with elderly people in Germany identified that polypharmacy is common among those in this population who have multimorbidity ^16^. It also showed that less than half (44.1%) of individuals without multimorbidity used polypharmacy, while the vast majority (75.1%) of individuals with multimorbidity used polypharmacy. However, when assessing association of polypharmacy with non-cancer mortality in individuals with or without multimorbidity, the estimates for polypharmacy (defined as 5-9 medications) were similar in both strata (HR 1.07 without multimorbidity and HR 1.14 with multimorbidity), while for hyperpolypharmacy (≥10 medications) there was a difference, although it was not significant (HR 1.42; 95%CI 0.57; 3.57) in individuals without multimorbidity and with multimorbidity (HR 0.51; 95%CI 0.11; 2.27). The authors suggest that the association between hyperpolypharmacy and non-oncological mortality might only be present in those elderly people without multimorbidity, which would be logical, as hyperpolypharmacy is not indicated for patients without multimorbidity ^16^. The polypharmacy prevalence we found (36.1%) demonstrates that it is a reality among the elderly. A study carried out in São Paulo found 36% polypharmacy prevalence among elderly people ^17^, while another study conducted in Florianópolis found 32% (95%CI 29.8; 34.3) ^18^. Similar results were also found in other countries, such as the study that evaluated the geographic distribution of polypharmacy in Europe, obtaining a variation ranging from 26.3% (95%CI 25.8; 26.8) to 39.9% (95%CI 39.3; 40.5) ^19^. Pharmacology presents peculiarities in elderly people, as changes occur both in pharmacokinetics, which affect the concentration and distribution of drugs, and also in pharmacodynamics, which cause changes in the effect of drugs on organs and tissues ^20^. Adverse reactions arise not only due to pharmacological changes, but, above all, due to drug and drug/disease interactions, making the process of caring for elderly people a challenge for health professionals ^21^. In order to reduce the risk of undesired consequences, efforts should be made to reduce the number years that older people live with polypharmacy ^22^. One methodology that has been applied is deprescribing, a method defined as the withdrawal of medications considered harmful or of little benefit, with the aim of controlling polypharmacy, improving outcomes and, consequently, quality of life ^23^. In the analysis stratified by age group, we found that polypharmacy prevalence increased the older the age group. Results of a study in Brazil in 2014 ^24^ partially differ from ours, as they found significantly higher polypharmacy prevalence among elderly people aged 70-79 years (22.0%). In that study, data from the National Survey of Access, Use and Promotion of Rational Use of Medication were used and polypharmacy prevalence stratified in the same age groups was assessed, being more wide-ranging, with representation of the five regions of Brazil. On the other hand, the authors only assessed use of medications for chronic diseases, linking the medications to prior diagnosis of the most prevalent chronic noncommunicable diseases among elderly people ^24^. In our study we assessed all medications used by elderly people, whether for chronic diseases or not. When assessing polypharmacy prevalence stratified by age group and sociodemographic characteristics, we found greater polypharmacy prevalence in females aged 60-79. This result can be attributed to the fact that women in this age group present consequences and symptoms of menopause ^25^. One must also consider the fact that women have more non-fatal health problems, take more care of themselves, are more alert to symptoms and pay greater attention to their health problems, using health services more and, consequently, using more medications ^26^. However, in the 80 and over age group, polypharmacy prevalence was higher among males. This can be explained by the fact that men seek medical care later or even ignore the symptoms of some diseases ^27^.

In relation to care in health services, higher polypharmacy prevalence was identified in private/ health insurance care services and polypharmacy was predominant in economic classes A/B and C. This suggests greater use of medications by the population with sufficient purchasing power to buy them and pay for health insurance or private services. The same reasoning can be applied to schooling, as the study showed higher polypharmacy prevalence among elderly people with higher schooling levels, suggesting that knowledge attracts greater professional possibilities, which, in turn, in addition to enabling greater health care, provide more knowledge about illnesses and self- care. Our results demonstrate a positive relationship between purchasing power and access to medications. These findings are in line with the study that described the sociodemographic profile of medication users in Brazil ^28^, which found greater use of medications among people belonging to the highest economic class, since use of medications depends on access and this may be conditioned to purchasing power in the absence of free of charge supply ^28^.

We found higher polypharmacy prevalence among people in the 70-79 age group who did not have a partner. A study shows that the presence of a spouse protects against health problems ^17^, leading to less medication use. In relation to work status, those who were not working, aged 60-69, had higher polypharmacy prevalence. Elderly people remaining on the job market depends on adequate health status ^17^.

When analyzing polypharmacy prevalence according to lifestyle, elderly people who did not smoke, were aged 70-79 years and those who had not consumed alcoholic beverages in the last 30 days, showed a higher prevalence. This result diverges from the expected, as it is believed that smoking tobacco and drinking alcohol lead to more illnesses and, consequently, use of more medications. However, use of more medications by elderly non-smokers who do not consume alcoholic beverages may be related to greater health care, using health services more frequently or even the fact that they have stopped smoking or consuming alcoholic

beverages due to use of medications. Polypharmacy prevalence was higher when self- perceived health was reported as poor or very poor ^29^ ^,^ ^30^. This result can be explained by the fact that as elderly people realize that their health is not as good as it could be, they try to resolve this by going to health services, receiving medication prescriptions or even self-medication.

The strengths of the study are related to the fact that it is a population-based study and its sample size. The findings have important practical implications for elderly people who use polypharmacy, specially when considering that it is a modifiable risk factor, as inappropriate use brings adverse health outcomes and increases the risk of death.

The main objective of the study was to characterize polypharmacy prevalence and its association with mortality among elderly people. We found that in a cohort of 1,451 elderly people, polypharmacy was associated with mortality and that there was no effect modification in the association of polypharmacy with mortality when assessing the interaction of age group and multimorbidity, indicating that association between polypharmacy and mortality did not vary depending on the age group of the elderly and the presence of multimorbidity did not affect this association.

## Data Availability

Access to the database and analysis codes, methods and other materials used in this research may be requested from the lead author or the corresponding author.
