# Supplementary material for: Prevalence of polypharmacy use and association with mortality: a cohort study of elderly people in Southern Brazil, 2014-2017
Source: Epidemiol Serv Saude. 2025 Apr 7;34:e20240081. doi: 10.1590/S2237-96222025v33e20240081.en (PMC11978280; doi:10.1590/S2237-96222025v33e20240081.en)
Supplement: Translation

# Translation: Prevalência do uso da polifarmácia e associação com mortalidade: estudo de coorte de pessoas idosas no Sul do Brasil, 2014-2017

**Authors:** Cristina Heloisa Müller, Andréa Dâmaso Bertoldi, Renata Moraes Bielemann, Karla Pereira Machado, Elaine Tomasi, Maria Cristina Gonzalez, Marysabel Pinto Telis Silveira

**Affiliations:** 1 Universidade Federal de Pelotas, Instituto de Fisiologia, Programa de Pós-Graduação Multicêntrico em Ciências Fisiológicas, Pelotas, RS, Brazil; 2 Universidade Federal de Pelotas, Faculdade de Medicina, Programa de Pós-Graduação em Epidemiologia, Pelotas, RS, Brasil; 3 Universidade Federal de Pelotas, Faculdade de Nutrição, Programa de Pós-Graduação em Nutrição e Alimentos, Pelotas, RS, Brasil

**Keywords:** Polimedicação, Idoso, Envelhecimento, Mortalidade, Estudos de coortes

## Abstract

**Objetivo:**

Caracterizar a prevalência do uso de polifarmácia e sua associação com mortalidade em pessoas idosas.

**Métodos:**

Coorte prospectiva (2014 a 2017), com indivíduos de 60 anos ou mais não institucionalizados, residentes em Pelotas, Rio Grande do Sul. A associação entre polifarmácia e mortalidade foi analisada com regressão de Cox. Foram calculadas as hazard ratios (HR) e seus respectivos intervalos de confiança de 95% (IC95%), seguindo o modelo de riscos proporcionais de Cox. A interação da faixa etária e multimorbidade foi considerada com p-valor&lt;0,100 como estatisticamente significativo.

**Resultados:**

A prevalência de polifarmácia foi 36,1% (IC95% 33,7; 38,6), sendo maior conforme maior a faixa etária (29,8% naqueles de 60-69 anos; 41,3% com 70-79 e 47,8%

na faixa etária de 80 anos ou mais). Na análise ajustada, o risco de mortalidade foi 62% maior entre pessoas idosas em uso de polifarmácia (HR 1,62; IC95% 1,10; 2,39), sem interação da faixa etária (p-valor 0,750) e multimorbidade (p-valor 0,312). Na análise de sobrevida, foi demonstrado que a probabilidade de sobrevida foi menor naquelas pessoas idosas com polifarmácia (85,6%).

**Conclusão:**

Identificada maior prevalência de polifarmácia conforme maior a faixa etária e pessoas idosas com polifarmácia apresentaram maior risco de mortalidade, independente da faixa etária e da presença de multimorbidade.

**Aspectos éticos**

A presente pesquisa respeitou os princípios éticos, obtendo os seguintes dados de aprovação:

Comitê de ética em pesquisa: Universidade Federal de Pelotas

Número do parecer: 472.357

Data de aprovação: 28/11/2013

Certificado de apresentação de apreciação ética: 54141716.0.0000.5317

Termo de consentimento: Obtido de todos os participantes antes da coleta.

## Introdução

O uso de medicamentos por pessoas idosas, apesar dos riscos, é de suma importância não só como estratégia farmacológica para diminuir o processo de degradação do envelhecimento, mas também no controle de doenças crônicas ^1^. Se, por um lado os indivíduos estão vivendo mais devido às descobertas de novos medicamentos, por outro, temos o risco potencial de interações farmacológicas e efeitos adversos pelo uso de múltiplos medicamentos, tornando-se uma das principais causas de admissão em serviços de saúde e um grande problema de saúde pública ^2^.

Novos estudos para garantir a segurança da farmacoterapia são fundamentais, sobretudo devido à complexidade entre o envelhecimento e a utilização de múltiplos medicamentos, bem como da inafastável correlação com o tempo de sobrevida conforme aumenta a idade. Tendo em vista que as pessoas idosas, conforme Censo Demográfico de 2022, representam importante parcela da população brasileira (15,8%) ^3^, e que o uso de medicamentos é mais frequente nesta faixa etária, este assunto torna-se de suma relevância.

O termo polifarmácia foi estudado para identificar e resumir as definições na literatura existente, em uma revisão sistemática, no período de 2000 a 2016 ^4^. Esta revisão destaca o desafio na definição frente à variabilidade no limite numérico, bem como as inconsistências em relação à duração da terapia e o uso de medicamentos sem prescrição ^4^. A maioria dos estudos define polifarmácia como o uso concomitante de cinco medicamentos ou mais ^5^ ^,^ ^6^. A limitação da literatura, assim como a comparação entre os estudos torna-se difícil devido à variação dos aspectos metodológicos, entre eles, a definição de polifarmácia, as faixas etárias e o tempo de seguimento.

Estudo de 18 anos de acompanhamento realizado na Inglaterra e no país de Gales relata a polifarmácia associada ao aumento da mortalidade em curto prazo em pessoas idosas, independente de sexo, idade, tabagismo, institucionalização, deficiência nas atividades da vida diária e condições de saúde. Registraram que não está bem elucidado se a polifarmácia é marcador de problemas de saúde ou risco independente de mortalidade, tendo sido descrita preditora de resultados adversos a medicamentos ^7^.

O objetivo deste trabalho foi caracterizar a prevalência do uso de polifarmácia e sua associação com mortalidade em pessoas idosas.

## Métodos

### Desenho do estudo

Estudo de coorte prospectiva (2014 a 2016 e 2017).

### Contexto

O estudo iniciou em 2014, sendo localizadas e entrevistadas em suas residências 1.451 pessoas idosas. No primeiro momento tratava-se de estudo transversal, em 2016 optou-se por dar continuidade, sendo denominado: “Estudo Longitudinal da Saúde do Idoso”- COMO VAI?”, iniciando-se a segunda etapa do acompanhamento com entrevistas telefônicas e domiciliares, sendo entrevistadas 1.161 pessoas idosas (Figura 1). Os telefonemas foram realizados em dife- rentes dias e horários, aqueles para os quais não foi possível realizar o contato telefônico foram buscados em seus endereços, com pelo menos quatro tentativas. Esse planejamento foi importante para a confirmação de informações básicas, permitindo identificar os óbitos no Sistema de Informação sobre Mortalidade.

**Figura 1:** Número de participantes em cada etapa do estudo. Pelotas, 2014-2017

### Participantes

Participaram indivíduos com 60 anos ou mais, não institucionalizados, residentes na zona urbana de Pelotas, Rio Grande do Sul.

### Variáveis

A variável de exposição principal foi polifarmácia, caracterizada como o uso de cinco ou mais medicamentos ^5^, sendo medida na avaliação inicial (2014). O desfecho foi mortalidade, o tempo de observação utilizado para estimá-lo foi da data da entrevista em 2014 até o óbito ou o fim do período de acompanhamento (2016/2017).

### Fonte de dados e mensuração

Para verificar o uso de medicamentos foram realizadas as perguntas: o(a) sr.(a) precisa tomar algum remédio de uso contínuo? e: o(a) sr.(a) usou algum remédio nos últimos 15 dias? O óbito foi considerado por todas as causas, sendo a informação fornecida nas entrevistas telefônicas ou domiciliares, por um parente ou vizinho e posteriormente conferida junto ao Setor de Vigilância Epidemiológica da Secretaria Municipal de Saúde de Pelotas, por meio do Sistema de Informação sobre Mortalidade e por certificação do óbito por documentos dos familiares. Quando houve recusa em participar desta etapa, assim como indivíduos que foram classificados como perda por falta de contato, foram considerados na análise como vivos devido à ausência de informação do óbito.

### Controle de viés

Para minimizar o viés de memória foram solicitadas receitas e embalagens e perguntou-se sobre os medicamentos de uso contínuo, além daqueles usados nos últimos 15 dias. As pessoas idosas perdidas no estudo foram consideradas proporcionais ao longo do tempo, sendo assim, considerada uma posição mais conservadora para cálculo das estimativas de HR. Ou seja, na medida pessoa-tempo em risco foi considerado até a metade do período total de acompanhamento, pois não havia conhecimento do momento exato entre uma visita e outra que o idoso se tornou perda. Foram adotadas estratégias para a devida identificação das mortes ocorridas entre os participantes.

### Tamanho do estudo 

O tamanho da amostra foi estimado para o consórcio de pesquisa realizado pelo programa de Pós-Graduação em Epidemiologia da Universidade Federal de Pelotas ^8^.

Em 2014 estimou-se uma maior amostra necessária para atingir os objetivos do estudo de cada mestrando, considerando acréscimo de 15% para fatores de confusão e 10% para perdas e recusas, além do efeito de delineamento amostral (1,5%), sendo de 1.649 ^9^. Para o trabalho de mestrado objetivando identificar o uso de medicamentos inapropriados para pessoas idosas o tamanho amostral total foi de 1.646 sujeitos ^9^ ^,^ ^10^.

O processo amostral foi realizado em dois estágios. Primeiramente foram escolhidos os conglomerados identificados no Censo de 2010 ^11^. Dos 488 setores censitários, 469 foram elegíveis, agrupando os que tinham pequeno número de pessoas idosas comparado aos demais. Para realizar o sorteio, os setores foram ordenados de acordo com a renda média, garantindo a inclusão de todos os bairros e com situações econômicas distintas. Cada setor continha informação do número de domicílios, pelo número inicial e número final, totalizando 107.152 domicílios. Baseado no Censode 2010, para incluir os 1.649 indivíduos foi necessário englobar 3.745 domicílios.

No segundo estágio ocorreu o sorteio dos setores para a seleção dos domicílios. Foram selecionados 31 domicílios por setor possibilitando a identificação de, no mínimo, 12 pessoas idosas em cada, implicando na inclusão de 133 setores censitários ^9^.

### Variáveis quantitativas

Nas variáveis independentes foram incluídos dados demográficos e socioeconômicos, de estilo de vida e relacionados às condições de saúde: faixa etária (em anos: 60-69; 70-79; 80 ou mais); raça/cor da pele observada (branca; não branca); sexo (masculino; feminino); escolaridade (em anos de estudo: nenhum; menos de 4; 4-10; 11 ou mais); classificação econômica de acordo Associação Brasileira de Empresas de Pesquisa ^12^ agrupada em (A/B; C; D/E); situação conjugal (casado/com companheiro(a); solteiro/separado, viúvo/sem companheiro(a)); e, situação laboral atual (trabalhando; não trabalhando).

As variáveis de estilo de vida incluíram tabagismo (não, nunca fumou; sim, fuma 1 ou + cigarro(s) por dia há mais de 1 mês; já fumou, mas parou de fumar) e consumo de bebida alcoólica nos últimos 30 dias (não; sim).

As variáveis relacionadas às condições de saúde foram: a) autopercepção de saúde, pela pergunta: Como você considera a sua saúde? (boa, muito boa; regular; ruim, muito ruim); b) multimorbidade (não; sim), definida como a presença de duas ou mais condições crônicas ^13^ coletada pela pergunta Algum médico ou profissional de saúde já disse que o(a) sr.(a) tem (...)? sendo listadas 28 doenças ou sintomas; c)atendimento no serviço de saúde (particular ou convênio/Sistema Único de Saúde;SUS;) pelas perguntas: onde o(a) sr.(a) consultou pela última vez, no último ano? e o atendimento, nesse último serviço de saúde, foi por foi por algum convênio, particular ou pelo SUS?.

### Métodos estatísticos

As análises estatísticas foram realizadas no sof-tware Stata versão 17 (StataCorp, College Station).Inicialmente foi realizada análise descritiva da amostra total e da prevalência de polifarmácia de acordo com variáveis sociodemográficas, de estilo de vida e de condições de saúde, utilizando os testes de qui-quadrado ou de tendência linear. Posteriormente analisada a prevalência de polifarmácia de acordo com as variáveis estudadas, estratificado por faixa etária, apresentando as proporções e os respectivos intervalos de confiança de 95% (IC95%), sendo utilizado o teste qui-quadrado.

Para avaliar o efeito da polifarmácia na mortalidade foram calculadas as hazard ratios (HR) e seus respectivos IC95%, seguindo o modelo de riscos proporcionais de Cox, utilizando regressão de Cox. O ajuste estatístico foi realizado incluindo a variável de exposição principal (polifarmácia) e o desfecho (mortalidade) baseado em modelo hierárquico com quatro níveis, sendo o nível 1 composto pelas variáveis sociodemográficas e econômicas, o 2 pelas variáveis de estilo de vida e o 3 as de condições de saúde. Mantidas no modelo final apenas as variáveis com p-valor <0,200. A seguir, foi realizada análise de sobrevida de Kaplan-Meier e o gráfico da função de risco cumulativo de acordo com o tempo para avaliar se os resultados poderiam ser influenciados pelo tempo entre os eventos. O teste Log-Rank foi empregado para comparação das funções de sobrevida entre indivíduos com polifarmácia e sem polifarmácia. As variáveis de faixa etária e multimorbidade foram avaliadas como modificadoras de efeito da associação entre polifarmácia e mortalidade, considerando p-valor <0,100 como estatisticamente significativo. Após o ajuste, foi testada a interação removendo a variável “faixa etária” do modelo para avaliar a possível influência da interação, logo, a variável foi reintegrada ao modelo ajustado. Foram obtidas as assinaturas no termo de consentimento livre, esclarecido aos indivíduos que participaram da pesquisa ou seus responsáveis, e garantida a confidencialidade dos dados. Nos casos de óbito, em 2016/2017, foi assinado pelos familiares ou informantes. Nas entrevistas telefônicas, o consentimento para responder o questionário foi verbal.

## Resultados

Em 2014 foram localizadas 1.844 pessoas idosas, após 393 perdas e recusas (21,3%), 1.451 foram incluídas. Dessas 1.451, 153 (10,5%) foram perdas e recusas, sendo localizadas no segundo acompanhamento (2016/2017), 1.298 pessoas idosas. Destas 1.161 foram entrevistadas, resultando em 137 (10,5%) perdas e recusas (Figura 1).

Na tabela 1 está descrita a análise da amostra to-tal de acordo com as variáveis sociodemográficas, de estilo de vida e de condições de saúde. A prevalência de polifarmácia na amostra geral foi de 36,1% (IC95% 33,7; 38,6), sendo maior entre os mais idosos (29,8% naqueles de 60-69 anos; 41,3% com 70-79 e 47,8% na faixa etária de 80 anos ou mais), nos de raça/cor de pele branca (37,7%), do sexo feminino (39,3%), que não estavam trabalhando (38,9%), ex-fumantes (39,5%), que não consumiram bebida alcoólica nos últimos 30 dias (38,4%), com pior autopercepção de saúde (56,1%), que tinham multimorbidade (37,4%) e com atendimento no serviço de saúde de forma particular ou convênio (42,1%) (Tabela 1).

**Tabela 1:** Descrição da amostra total e prevalência de polifarmácia de acordo com variáveis sociodemográficas, de estilo de vida e de condições de saúde. Pelotas, 2014-2017 (n=1.451)

Variável	Amostra	Polifarmácia	Polifarmácia
	n (%)	% (95%CI)	p-valor
**Faixa etária (anos)**_a_			<0.001
60-69	756 (52.3)	29.8 (26.6; 33.1)	
70-79	460 (31.8)	41.3 (36.9; 45.9)	
≥80 230	(15.9) 47.8	(41.4; 54.3)	
Raça/cor da pele_a_			0.014
Branca	1,211 (83.7)	37.7 (35.0; 40.4)	
Não branca	236 (16.3)	29.2 (23.8; 35.4)	
**Sexo**			0.001
Masculino	537 (37.0)	30.9 (27.1; 35.0)	
Feminino	914 (63.0)	39.3 (36.2; 42.5)	
**Escolaridade (anos)**_a_			0.856
Nenhuma	196 (13.6)	35.7 (29.3; 42.7)	
< 4	337 (23.5)	37.7 (32.7; 43.0)	
4-10	588 (40.9)	36.6 (32.8; 40.5)	
11 ou mais	316 (22.0)	34.5 (29.4; 39.9)	
**Classe econômica**_a_			0.065
A/B	483 (35.2)	36.0 (31.9; 40.4)	
C	720 (52.5)	37.4 (33.9; 41.0)	
D/E	169 (12.3)	27.8 (21.5; 35.1)	
**Situação conjugal**_a_			0.130
Casado/com companheiro(a)	763 (52.7)	34.5 (31.2; 37.9)	
Solteiro/separado/viúvo/sem companheiro(a)	684 (47.3)	38.3 (34.7; 42.0)	
**Situação laboral**_a_			<0.001
Trabalhando	264 (19.6)	24.2 (19.4; 29.8)	
Não trabalhando	1,084 (80.4)	38.9 (36.1; 41.9)	
**Tabagismo**_a_			<0.001
Não, nunca fumou	781 (54.0)	37.5 (34.2; 41.0)	
Sim, fuma 1 ou + cigarro(s) por dia há mais de 1 mês	182 (12.6)	22.5 (17.0; 29.2)	
Já fumou, mas parou de fumar	483 (33.4)	39.5 (35.3; 44.0)	
**Consumo de bebida alcoólica**_a_			0.002
Não	1,138 (78.8)	38.4 (35.6; 41.3)	
Sim	307 (21.3)	28.7 (23.9; 34.0)	
**Autopercepção de saúde**_a_			<0.001
Boa/muito boa	765 (53.1)	26.1 (23.1; 29.4)	
Regular	545 (37.8)	45.5 (41.4; 49.7)	
Ruim/muito ruim	132 (9.2)	56.1 (47.4; 64.3)	
**Multimorbidade**_a_			<0.001
Não	89 (6.7)	6.7 (3.0; 14.3)	
Sim	1,250 (93.4)	37.4 (34.8; 40.2)	
**Atendimento no serviço de saúde**_a_			0.029
Particular ou convênio	812 (63.4)	42.1 (38.8; 45.6)	
Sistema Único de Saúde	468 (36.6)	35.9 (31.7; 40.4)	

Nota: aVariáveis com dados faltantes: faixa etária (n=5), raça/cor da pele (n=4), escolaridade (n=14), classe econômica (n=79), situação conjugal (n=4), situação laboral (n=103), tabagismo (n=5), consumo de bebida alcoólica (n=6), autopercepção de saúde (n=9), multimorbidade (n=112), atendimento no serviço de saúde (n=171).

A Tabela 2 descreve a prevalência de polifarmácia com seus respectivos intervalos de confiança, estratificada por faixa etária, para cada uma das variáveis estudadas. Aos 60-69 anos a prevalência de polifarmácia foi maior no sexo feminino (33,4%), na classe socioeconômica C (33,2%), naqueles que não estavam trabalhando (32,9%), que não consumiram bebida alcoólica nos últimos 30 dias (31,7%), que tinham autopercepção de saúde ruim ou muito ruim (56,5%) e que apresentavam multimorbidade (31,8%).

Naqueles com 70-79 anos, a prevalência de polifarmácia foi maior no sexo feminino (45,8%), com autopercepção de saúde relatada como ruim ou muito ruim (61,4%) e que tinham multimorbidade (42,2%).

Também foi maior nos que vivem sem companheiro (46,4%), que nunca fumaram (44,8%) e com atendimento no serviço de saúde particular ou convênio (49,6%) (Tabela 2).

**Tabela 2:** Prevalência de polifarmácia de acordo com variáveis sociodemográficas, de estilo de vida e de condições de saúde, estratificado por faixa etária. Pelotas, 2014-2017 (n=1.415)

Variáveis	60-69 anos		70-79 ano		≥80 anos	
	% (95%CI)	p-valor		p-valor		p-valor
**Raça/cor da pele**		0.056		0.578		0.043
Branca						
Não branca						
**Sexo**		0.005		0.011		0.378
Masculino	23.9 (19.3; 29.2)		33.7 (27.0; 41.2)		52.0 (40.6; 63.2)	
Feminino	33.4 (29.3; 37.8)		45.8 (40.1; 51.6)		45.8 (38.1; 53.8)	
**Escolaridade (anos)**		0.482		0.648		0.043
Nenhuma	21.7 (12.9; 34.1)		46.1 (35.1; 57.4)		36.7 (25.3; 49.7)	
< 4	32.5 (25.6; 40.3)		42.9 (34.2; 52.0)		41.0 (29.2; 53.9)	
4-10	30.2 (25.5; 35.3)		40.3 (33.4; 47.7)		58.0 (45.9; 69.2)	
11 ou mais	29.6 (23.6; 36.4)		36.6 (26.8; 47.7)		56.8 (40.1; 72.0)	
**Classe econômica**_a_		0.007		0.579		0.166
A/B	27.4 (22.4; 33.0)		43.0 (34.6; 51.8)		55.7 (44.5; 66.4)	
C	33.2 (28.6; 38.2)		41.1 (35.0; 47.3)		43.3 (34.0; 53.0)	
D/E	15.5 (8.7; 26.1)		35.0 (23.9; 48.1)		40.5 (25.7; 57.3)	
**Situação conjugal**		0.509		0.042		0.398
Casado/com companheiro(a)	30.7 (26.6; 35.1)		37.1 (31.3; 43.2)		52.5 (39.8; 64.8)	
Solteiro/separado/viúvo/sem companheiro(a)	28.4 (23.6; 33.8)		46.4 (39.7; 53.2)		46.2 (38.7; 53.8)	
**Situação laboral**		0.003		0.081		0.453
Trabalhando	21.6 (16.6; 27.7)		28.2 (16.0; 44.7)		58.3 (27.8; 83.6)	
Não trabalhando	32.9 (28.9; 37.1)		42.6 (37.8; 47.6)		47.2 (40.2; 54.2)	
**Tabagismo**		0.060		0.007		0.289
Não, nunca fumou	29.4 (25.0; 34.3)		44.8 (38.8; 50.9)		45.8 (37.8; 54.1)	
Sim, fuma 1 ou + cigarro(s) por dia há mais de 1 mês	22.2 (15.8; 30.4)		19.0 (9.6; 34.2)		35.7 (14.3; 65.0)	
Já fumou, mas parou de fumar	34.0 (28.4; 40.1)		41.5 (34.1; 49.4)		54.9 (43.1; 66.2)	
**Consumo de bebida alcoólica**		0.046		0.272		0.181
Não	31.7 (28.0; 35.7)		42.5 (37.6; 47.6)		49.8 (42.8; 56.7)	
Sim	24.1 (18.5; 30.7)		36.0 (26.5; 46.8)		36.7 (21.1; 55.7)	
**Autopercepção de saúde**		<0.001		<0.001		<0.001
Boa/muito boa	22.0 (18.4; 26.2)		28.2 (22.7; 34.4)		40.0 (30.8; 50.0)	
Regular	36.9 (31.1; 43.0)		51.9 (44.7; 59.0)		55.9 (46.0; 65.3)	
Ruim/muito ruim	56.5 (43.7; 68.4)		61.4 (46.0; 74.8)		48.0 (28.8; 67.8)	
**Multimorbidade**_b_		<0.001		0.001		0.021
Não	6.9 (2.6; 17.3)		4.8 (0.6; 29.8)		10.0 (1.0; 54.7)	
Sim	31.8 (28.3; 35.4)		42.2 (37.5; 47.0)		47.5 (40.2; 54.9)	
**Atendimento no serviço de saúde**		0.251		0.006		0.013
Particular ou convênio	31.9 (27.4; 36.7)		49.6 (43.7; 55.6)		55.2 (47.2; 62.9)	
Sistema Único de Saúde	36.2 (30.6; 42.1)		35.5 (28.0; 43.8)		35.7 (24.1; 49.3)	

Nos idosos com 80 anos ou mais a prevalência de polifarmácia foi maior nos de raça/cor de pele branca (51,1%), com 11 ou mais anos de estudo (56,8%), com multimorbidade (47,5%) e atendimento no serviço de saúde particular ou convênio (55,2%) (Tabela 2).

Foram identificados 145 óbitos (10%). O tempo de acompanhamento foi de 2,6 (+/-0,5) anos. Os modelos bruto e ajustado de regressão de riscos proporcionais de Cox são apresentados na Tabela 3. A polifarmácia permaneceu como fator de risco para óbito após ajuste para sexo, faixa etária, escolaridade, situação conjugal, situação laboral, tabagismo, consumo de bebida alcoólica e autopercepção de saúde, aumentando o risco de mortalidade em 62% (HR 1,62; IC95% 1,10; 2,39). Não foi observado indício de modificação de efeito para faixa etária (p-valor 0,750) nem para multimorbidade (p-valor 0,312).

**Tabela 3:** Hazard hatios (HR) bruta e ajustada e intervalo de confiança (IC95%) da polifarmácia pelas variáveis do estudo Pelotas, 2014-2017 (n=1.451)

Variável	Análise bruta		Análise ajustada	
	HR (IC95%)	p-valor	HR (IC95%)	p-valor
Polifarmácia				
Toda amostra		<0,001		0,014
Não	1,00		1,00	
Sim	2,07 (1,48; 2,89)		1,62 (1,10; 2,39)	

A Figura 2 mostra que ambas as curvas de sobrevivência decaem com o passar do tempo, ou seja, a probabilidade de sobrevida diminuiu conforme maior faixa etária, tanto para pessoas idosas com polifarmácia como para aquelas sem polifarmácia. Porém, a probabi-

lidade de sobrevida, após três anos, dos indivíduos com polifarmácia na avaliação inicial foi menor (85,6%) que nos indivíduos sem polifarmácia (92,6%). Não houve diferença significativa na análise bruta (p-valor 0,055, teste de log-rank) porém a análise ajustada apresentou diferença (p-valor 0,014).

**Figura 2:** Probabilidade de sobrevivência cumulativa de acordo com polifarmácia em pessoas idosas, Pelotas, 2014-2017 (n=1.451)

## Discussão

Os resultados demonstram que a polifarmácia está independentemente associada à mortalidade em pessoas idosas. Hipoteticamente, acreditava-se que o risco de mortalidade com polifarmácia seria maior quanto maior a faixa etária, pois estariam mais propensos a apresentar mais doenças ^14^ e, consequentemente, usar mais medicamentos. Entretanto, a polifarmácia diminuiu a probabilidade de sobrevida independente da faixa etária. Esse resultado é semelhante ao estudo anterior que analisou a mortalidade associada à polifarmácia. A sobrevida de pessoas idosas expostas à polifarmácia foi avaliada no Brasil no período de 2006-2010 ^15^, demonstrando que a probabilidade de sobrevida após cinco anos dos indivíduos com polifarmácia foi de 77,2%, enquanto nos indivíduos sem polifarmácia foi de 85,5% (p-valor <0,001). Igualmente neste estudo, a curva de sobrevivência mostra que a probabilidade de óbito foi maior nas pessoas idosas com polifarmáciaao longo do acompanhamento ^15^. Uma das possíveis limitações do nosso estudo é o tempo de acompanhamento relativamente curto. Contudo, o estudo de coorte continua, o que permitirá avaliar a mortalidade em tempo maior no futuro. Outra limitação é a possível ocorrência de viés de memória, pois era necessário que recordassem os medicamentos utilizados nos últimos 15 dias. Este viés foi minimizado solicitando receitas e embalagens e perguntando também sobre os medicamentos de uso contínuo.

No presente estudo, a multimorbidade não modificou o efeito da associação entre polifarmácia e mortalidade. A prevalência de polifarmácia foi significativamente maior com a presença de multimorbidade em todas as faixas etárias, aumentando conforme o avançar da idade.

Estudo de coorte com pessoas idosas na Alemanha identificou que a polifarmácia é comum nessa população com multimorbidade ^16^. Também evidenciou que menos da metade (44,1%) dos indivíduos sem multi-morbidade fazia uso de polifarmácia, enquanto a grande maioria (75,1%) dos indivíduos com multimorbidade fazia uso de polifarmácia. Contudo, quando avaliada a associação de polifarmácia com mortalidade não oncológica em indivíduos com ou sem multimorbidade, as estimativas para polifarmácia (definida como 5-9 medicamentos) foram semelhantes nos dois estratos (HR 1,07 sem multimorbidade e HR 1,14 com), enquanto que para a hiperpolifarmácia ( ≥10 medicamentos) houve diferença, porém, não significativa (HR 1,42; IC95% 0,57; 3,57) nos indivíduos sem multimorbidade e com multimorbidade (HR 0,51; IC95% 0,11; 2,27). Os autores sugerem que associação de hiperpolifarmácia e mortalidade não oncológica poderia estar presente apenas naquelas pessoas idosas sem multimorbidade, o que seria lógico, pois pacientes sem multimorbidade não têm indicação de hiperpolifarmácia ^16^.

A prevalência de polifarmácia encontrada (36,1%) demonstra que ela é uma realidade nesta população. Estudo realizado em São Paulo encontrou 36% de polifarmácia entre pessoas idosas ^17^, outro realizado em Florianópolis, 32% (IC95% 29,8; 34,3) ^18^. Resultados semelhantes também foram encontrados em outros países, como o estudo que avaliou a distribuição geográfica da polifarmácia na Europa, obtendo variação de 26,3% (IC95%25,8; 26,8) a 39,9% (IC95% 39,3; 40,5) (19).

A farmacologia apresenta peculiaridades em pessoas idosas, pois ocorrem alterações tanto na farmacocinética, que afetam a concentração e distribuição dos fármacos, como na farmacodinâmica, que provocam a alteração no efeito dos fármacos nos órgãos e tecidos ^20^. As reações adversas surgem não só pelas alterações farmacológicas, mas, sobretudo, devido às interações medicamentosas e medicamento/doença, tornando o processo do cuidar da pessoa idosa um desafio para os profissionais da saúde ^21^.

Para diminuir o risco de consequências indesejadas, esforços devem ser realizados para diminuir os anos que pessoas idosas vivem com polifarmácia ^22^. Uma metodologia que vem sendo aplicada é a desprescrição, método definido como a retirada de medicamentos considerados prejudiciais ou pouco benéficos, com o objetivo de controlar a polifarmácia, melhorar os desfechos e, consequentemente, a qualidade de vida ^23^.

Na análise estratificada por faixa etária foi observado que houve aumento da prevalência de polifarmácia conforme maior faixa etária.

Resultados de estudo no Brasil em 2014 ^24^ diferem parcialmente dos nossos, pois encontraram prevalência de polifarmácia significativamente maior entre pessoas idosas com 70-79 anos (22,0%). No referido estudo foram utilizados dados da Pesquisa Nacional de Acesso, Utilização e Promoção do Uso Racional de Medicamentos e foi avaliada a prevalência de polifarmácia estratificada nas mesmas faixas etárias, sendo mais abrangente, com representatividade das cinco regiões brasileiras. Por outro lado, os autores avaliaram somente a utilização de medicamentos de uso crônico, vinculando os medicamentos a um diagnóstico prévio referido das Doenças Crônicas Não Transmissíveis mais prevalentes entre as pessoas idosas ^24^. No nosso estudo avaliamos todos os medicamentos utilizados pelas pessoas idosas, sejam de uso crônico ou não. 

Ao avaliar a prevalência de polifarmácia estratificada por faixa etária e por características sociodemográficas, observamos maior prevalência de polifarmácia no sexo feminino com 60-79 anos. O resultado pode ser atribuído ao fato de que mulheres nessa faixa etária apresentam consequências e sintomas da menopausa ^25^. Também deve-se considerar o fato de que mulheres têm mais problemas de saúde não fatais, se cuidam mais, estão mais alertas aos sintomas e dão maior atenção aos seus problemas de saúde, procurando mais os serviços de saúde e, consequentemente, fazendo uso de mais medicamentos ^26^. Entretanto, na faixa etária de 80 anos ou mais, a prevalência de polifarmácia foi maior no sexo masculino. Isto pode ser explicado pelo fato de que homens procuram por cuidados médicos mais tardiamente ou até mesmo ignoram os sintomas de algumas doenças ^27^.

Em relação aos atendimentos nos serviços de saúde foi identificada maior prevalência de polifarmácia em atendimentos particular ou convênio e houve predominância de polifarmácia nas classes econômicas A/B e C. Isto sugere maior uso de medicamentos pela população com poder aquisitivo suficiente para compra, contratação de convênio ou pagamento privado. O mesmo raciocínio pode ser realizado com a escolaridade, pois o estudo apresentou maior prevalência de polifarmácia nas pessoas idosas com maior escolaridade, sugerindo que o conhecimento atrai maiores possibilidades profissionais, que, por sua vez, além de permitirem maior cuidado com a saúde, proporcionam mais conhecimento sobre doenças e autocuidado. Nossos resultados demonstram relação positiva entre o poder econômico e o acesso a medicamentos. Estes achados vão ao encontro do estudo que descreveu o perfil sociodemográfico dos usuários de medicamentos no Brasil ^28^, o qual encontrou maior utilização de medicamentos em pessoas pertencentes à maior classe econômica, visto que o uso dos medicamentos depende do acesso e este pode estar condicionado ao poder de compra na falta de fornecimento gratuito ^28^.

As pessoas idosas na faixa etária de 70-79 anos que não tinham companheiro apresentaram maior prevalência de polifarmácia. Estudo demonstra que a presença de cônjuge é protetora de agravos ^17^, levando a menor uso de medicamentos. Em relação à situação laboral, apresentaram maior prevalência de polifarmácia aqueles que não estavam trabalhando, com 60-69 anos. A permanência do idoso no mercado de trabalho depende de um estado de saúde adequado ^17^.

Quando analisada a prevalência de polifarmácia de acordo com o estilo de vida, as pessoas idosas que não fumavam, com 70-79 anos e as que não tinham consumido bebidas alcoólicas nos últimos 30 dias, apresentaram maior prevalência. O resultado diverge do senso comum, pois se imagina que o tabagismo e o álcool levariam ao acometimento de mais doenças e, consequentemente, do uso de mais medicamentos. Entretanto, o uso de mais medicamentos por pessoas idosas não fumantes e que não consomem bebida alcoólica pode estar relacionado ao maior cuidado com a saúde, à busca por serviços de saúde com maior frequência ou até mesmo que deixaram de fumar ou consumir bebida alcoólica devido ao uso de medicamentos.

A prevalência de polifarmácia foi maior quando a autopercepção de saúde foi declarada ruim ou muito ruim ^29^ ^,^ ^30^. Esse resultado pode ser justificado pelo fato de que à medida que o idoso percebe que sua saúde não está adequada procura solucioná-la, indo a serviços de saúde, recebendo prescrições de medicamentos ou até mesmo fazendo automedicação.

Os pontos fortes do estudo relacionam-se com o fato de ser um estudo de base populacional e seu tamanho amostral. As descobertas têm importantes implicações práticas voltadas às pessoas idosas que fazem uso de polifarmácia, sobretudo quando considerado que se trata de um fator de risco modificável, já que o uso de forma inadequada traz resultados adversos à saúde e aumenta o risco de morte.

O objetivo principal foi caracterizar a prevalência da polifarmácia e sua associação com mortalidade entre pessoas idosas. Mostrou-se que em uma coorte de 1.451 pessoas idosas a polifarmácia esteve associada à mortalidade e que não há modificação do efeito na associação de polifarmácia com mortalidade quando avaliada a interação da faixa etária e multimorbidade, indicando que a associação entre polifarmácia e mortalidade não variou conforme a faixa etária das pessoas idosas e a presença de multimorbidade não afetou esta associação.

## Data Availability

O acesso ao banco de dados e os códigos de análise, métodos e outros materiais utilizados na pesquisa poderão ser solicitados à autora coordenadora geral da pesquisa ou a autora correspondente.
